# Interpersonal Violence-Related Facial Fractures: 12-Year Trends and Surgical Outcomes in a Southern European Level-I Trauma Centre

**DOI:** 10.3390/medicina61081443

**Published:** 2025-08-11

**Authors:** Giulio Cirignaco, Lisa Catarzi, Gabriele Monarchi, Umberto Committeri, Andrea Frosolini, Lucrezia Togni, Marco Mascitti, Paolo Balercia, Andrea Santarelli, Giuseppe Consorti

**Affiliations:** 1 Department of Medicine, Section of Maxillo-Facial Surgery, University of Siena, Viale Bracci, 53100 Siena, Italy; lisa.catarzi@gmail.com (L.C.); gabriele.monarchi@gmail.com (G.M.); andreafrosolini@gmail.com (A.F.); 2 Division of Maxillofacial Surgery, Department of Neurological Sciences, Marche University Hospitals-Umberto I, 60126 Ancona, Italy; paolo.balercia@ospedaliriuniti.marche.it; 3 Division of Maxillofacial Surgery, “Santa Maria” Hospital, V.le Tristano di Joannuccio, 05100 Terni, Italy; umbertocommitteri@gmail.com; 4 Department of Clinical Specialistic and Dental Sciences, Marche Polytechnic University, 60126 Ancona, Italy; togni.lucrezia@gmail.com (L.T.); m.mascitti@staff.univpm.it (M.M.); andrea.santarelli@staff.univpm.it (A.S.)

**Keywords:** interpersonal violence, maxillofacial trauma, facial fractures, epidemiology, facial injury severity score (FISS)

## Abstract

*Background and Objectives:* Interpersonal violence (IPV) has overtaken road traffic collisions as a leading cause of facial fractures, yet regional data from Southern Europe are limited. *Materials and Methods:* We retrospectively reviewed all adults (≥18 y) treated between 1 January 2011 and 31 December 2022 for radiologically confirmed IPV-related facial fractures. Recorded variables were demographics, AO-CMF (Arbeitsgemeinschaft für Osteosynthesefragen—Craniomaxillofacial) fracture site, Facial Injury Severity Score (FISS), presence of facial soft-tissue wounds, treatment modality, and length of stay; associations between variables were explored. *Results:* A total of 224 victims were identified; 94% were men (median age 26 y, IQR 22–34). The mandible was the most frequently involved bone (42%), followed by the orbit (25%); 14% sustained fractures at multiple sites. Facial soft-tissue wounds occurred in 9% of cases, three-quarters of which were associated with mandibular injury (*p* = 0.005). The median FISS was 2 and was higher in males, patients > 34 y, those with multiple fractures, and those with wounds (all *p* < 0.05). FISS showed a weak positive correlation with hospital stay (r = 0.23), which averaged 4.1 ± 1.6 days. Open reduction and internal fixation were required in 78% of patients, most often 24–72 h after admission. Annual IPV-related admissions remained stable throughout the 12-year period. *Conclusions*: IPV in this region consistently injures young men, with the mandible and orbit most at risk. FISS is a practical bedside indicator of resource use. The unchanging incidence—likely underestimated because isolated nasal fractures and minor injuries are often managed outside maxillofacial services or never reported—highlights the urgency of targeted prevention programs, routine screening, and streamlined multidisciplinary pathways.

## 1. Introduction

Maxillofacial trauma and associated fractures exhibit different etiological patterns worldwide, depending on variables such as socioeconomic status, geographic location, and temporal factors in the area considered [1]. Consequently, both the etiology and severity of facial fractures differ across populations and frequently coexist with injuries to other body regions, resulting in distinct demographic profiles [2]. Given this variability, a region-specific approach is crucial for devising targeted preventive and clinical strategies, as maxillofacial fractures remain a major public health challenge worldwide, accounting for up to one-fifth of trauma admissions in specialized centers [3,4,5,6,7,8,9]. Large epidemiological series consistently show that the mandible is the single most commonly fractured facial bone, representing 22–42% of cases across continents.

The pattern of injury is highly dependent on geography, socioeconomic status, and legislation. In many low- and middle-income countries, RTAs continue to dominate, with figures exceeding 60% of all facial injuries [4]; by contrast, recent Italian and other European cohorts report a progressive rise in assaults and interpersonal violence, especially among young males. Reviews of domestic violence victims further confirm the face as a primary target, with the pooled prevalence of dento-maxillofacial injury approaching 30% [10,11,12,13].

At the national level, the picture is broadly similar but shows some notable differences. The European Maxillofacial Trauma (EURMAT) collaboration, a prospective study that pooled data from more than a dozen Italian maxillofacial units, attributed ≈ 27% of all facial fractures to interpersonal violence; the mandible (about 38%) and the zygomaticomaxillary complex (≈29%) were the most frequently involved sites, whereas orbital fractures accounted for only 14% of cases [14,15]. A single-center series from Turin, in northern Italy, analyzing 711 assault victims reported a comparable predominance of mandibular fractures (34%) but an even lower proportion of orbital injuries (9%) [16].

This type of involvement suggests that local assault dynamics like clenched-fist blows directed to the mid-face during nightlife-related altercations may differ from those recorded elsewhere in the country.

Management strategies are likewise consistent nationwide: both EURMAT and other cohorts document that open reduction and internal fixation (ORIF) is required in roughly two-thirds to four-fifths of IPV-related fractures, with most interventions performed within the first 72 h. Reported short-term outcomes are favorable, but multicenter audits highlight infection, malocclusion, and infra-orbital nerve dysfunction as the commonest complications, underscoring the need for timely surgery and strict postoperative follow-up [15,17,18,19].

Although interpersonal violence (IPV) is a well-documented cause of maxillofacial trauma, it remains underexplored in certain areas. IPV-related injuries not only result in physical harm but also carry profound emotional, social, and economic implications, emphasizing the importance of early detection and prevention. Victims of IPV commonly suffer head and face injuries. As IPV is typically related to young people, it has a great economic impact on both preventive efforts and the eventual psychological distress/disability reported [15]. The face is often the target in violent situations, owing to its lack of protection in daily life, exposure, and accessibility, leading to a high incidence of maxillofacial fractures. Research has also shown a connection between facial trauma and consequent low self-esteem and shame [10]. This vulnerability highlights the critical role of public health strategies in addressing the root causes of IPV.

The World Health Organization (WHO) defines violence as ‘The intentional use of physical force or power, threatened or actual, against oneself, another person, or against a group or community, that either results in or has a high likelihood of resulting in injury, death, psychological harm, maldevelopment, or deprivation’ [20]. WHO members formed the Violence Prevention Alliance with the belief that public health principles serve as an effective foundation for exploring and addressing the causes and impacts of violence while also working to prevent it through early intervention programs and policy measures [21]. Understanding the local dimensions of IPV also remains vital. Regional epidemiological data not only contribute to global knowledge but also guide resource allocation and tailored interventions that can mitigate the physical, psychological, and socioeconomic burdens of these injuries. The 2030 agenda places violence as a top priority, within an overarching goal to ‘promote peaceful and inclusive societies for sustainable development’ [21]. This agenda underscores the importance of regional data, such as those provided by this study.

During the past decades, causes and injury patterns have shifted, with IPV and assault emerging as major contributors to maxillofacial fractures globally, surpassing road traffic accidents—which have been mitigated by stricter legislation, better road conditions, and increased safety measures [1,14]. Meanwhile, IPV remains a persistent and deeply rooted issue, requiring continued efforts to develop effective societal and healthcare responses. In 2019, injuries overall took the lives of 4.4 million people worldwide, with 11% of all deaths connected to IPV [22].

Facial fractures associated with assault are often underreported and challenging to assess, largely owing to their frequent correlation with illicit activities and eventual consequences with law enforcement agencies [15]. Detailed analysis of these epidemiological, demographical, and geographical patterns could provide useful data to allow the development of more effective health policies and preventive measures to reduce the occurrence of aggression and related hospital admissions, thus reducing the impact on the Italian National Health Service (SSN), which has been subjected to significant reductions in financial allocations over the years [23,24].

Accordingly, the purpose of this study is to investigate the epidemiological and clinical characteristics of IPV-related maxillofacial trauma in a single center in central Italy, filling a gap in current regional data. In this context, our retrospective review examines the demographics, fracture patterns, and treatment outcomes of IPV-related cases. By correlating these data with metrics such as the Facial Injury Severity Score (FISS) and length of stay, we aim to identify critical areas for intervention in both clinical management and preventive strategies. Ultimately, this research underscores the importance of integrating public health principles with clinical practice to address the multifaceted challenges posed by IPV-related maxillofacial trauma.

## 2. Materials and Methods

### 2.1. Study Design and Sample

This study followed an observational design and used a hospital database for the analysis of patients admitted for maxillofacial trauma at the Maxillofacial Surgery Department in the University Hospital of Marche, Ancona, Italy, over a period from 1 January 2011 to 31 December 2022.

This is the only maxillofacial surgery unit in the entire region, with an area of 9365.86 km^2^ and a population of approximately 1,483,660 inhabitants (756,054 females and 727,606 males). The inclusion criteria were patients admitted for maxillofacial trauma with an associated fracture; ‘aggression’ recorded as the cause of trauma; and radiological examination confirming the clinical diagnosis of the fracture. The exclusion criteria were as follows: patients with incomplete data records; patients with isolated cranial vault fractures (i.e., without maxillofacial involvement) or injuries classified as self-inflicted (e.g., suicide attempts); and patients under 18 years old. Given the observational nature of the study, we did not perform any formal sample size calculation beforehand.

### 2.2. Variables and Data Collection Methods

For each patient in the database, we collected standardized data, including sex, age, cause and type of fracture, date of trauma, presence of facial soft-tissue wounds, severity of facial fractures (i.e., FISS assessment), associated injuries, and length of hospital stay. The anatomical fracture location was assessed using computed tomography scans performed during the diagnostic evaluation in the emergency department of our hospital or external facilities. Isolated nasal fractures are routinely managed by the Ear, Nose, and Throat (ENT) department in our region and were therefore only included when accompanied by at least one additional maxillofacial fracture.

The fractures were classified according to the AO-CMF criteria (Arbeitsgemeinschaft für Osteosynthesefragen—Craniomaxillofacial) [25,26,27] into the following categories: frontal sinus, orbit, nose, naso–orbital–ethmoidal complex, zygomaticomaxillary complex (or complex oral maxillofacial zone, zygomaticomaxillary complex), maxilla, and mandible. Each fracture was further classified according to its respective anatomical location, as defined by the AO classification system.

Maxillary fractures were divided by using the classification first introduced by Le Fort [28] into type I, II, and III. Le Fort I (horizontal) fractures separate the tooth-bearing maxilla and hard palate from the mid-face. The fracture line runs above the alveolus, crosses the lateral nasal wall, and terminates at the pterygoid plates. Le Fort II (pyramidal) fractures disrupt the zygomaticomaxillary and nasofrontal sutures, the frontal sinus floor, and the pterygoid processes, producing a mobile mid-facial “pyramid” and discontinuity of the inferomedial orbital rim. Damage to the medial maxillary buttress at this level explains the frequent epistaxis, cerebrospinal fluid rhinorrhea, or lacrimal apparatus injury seen in these patients. Le fort III fractures, known also as cranio-facial disjunction fractures, extend through the nasal bones; medial, inferior, and lateral orbital walls; pterygoid processes; and zygomatic arches, completely detaching the mid-face from the cranial base [29].

Facial soft-tissue wounds, when present, were evaluated based on their size and classified as either ≤10 cm or >10 cm in length.

### 2.3. International Classification of Diseases-10 Coding of Injuries

For each case, we assigned an International Classification of Diseases, 10th Revision (ICD-10) code [30]. Facial fractures fell under “Injuries to the head” (S00–S09) and were recorded as follows:

• S02.3 Fracture of orbital floor;
• S02.4 Fracture of malar and maxillary bones (zygomaticomaxillary complex, Le Fort);
• S02.6 Fracture of mandible (angle, condyle, symphysis, body, parasymphysis);
• S02.71 Multiple fractures involving skull and facial bones;
• S02.8 Other specified fractures of skull and facial bones (frontal-sinus fractures).

Concomitant soft-tissue injuries were coded as “Open wounds of head” (S01), most commonly:

• S01.4 Open wound of cheek and temporomandibular area;
• S01.5 Open wound of lips and oral cavity.

The ICD-10 categories are used solely for epidemiological comparison; the fracture morphology for surgical planning remained based on the AO-CMF system described above.

### 2.4. Facial Injury Severity Score (FISS)

The severity of each patient’s facial trauma was quantified with the Facial Injury Severity Score (FISS) [31,32,33]. The score assigns weighted points to every osseous or soft-tissue lesion: one point for fractures of the nasal bones, orbital rim/floor, zygomaticomaxillary complex, or palatal vault; two points for maxillary or Le Fort fractures; three points for isolated mandibular fractures; and four points for injuries of the frontal sinus or anterior cranial fossa. A deep soft-tissue laceration > 10 cm adds one additional point. The values are summed to yield a continuous score ranging from 0 to 15, where higher figures indicate more complex injuries.

To address potential sources of bias, we had all clinical data reviewed independently by two investigators (G.C. and G.C.). Efforts were made to ensure the accuracy of the reported variables by cross-checking medical and radiological reports. Misclassification bias was minimized by using standardized AO-CMF criteria and validated tools, such as FISS. Selection bias was reduced by including all eligible patients admitted during the study period, based on predefined inclusion and exclusion criteria. All surgical procedures were performed exclusively by experienced maxillofacial surgeons, ensuring consistency in the treatment approach and minimizing variability in outcomes associated with trainees or less experienced operators [34].

### 2.5. Statistical Analysis

Quantitative variables were reported with mean ± standard deviations (SDs), as well as median and interquartile ranges (first and third quartiles, Q1 and Q3), in descriptive tables. Their distribution was assessed using the Kolmogorov–Smirnov test, and non-parametric tests were applied in the case of asymmetrical distribution. We compared two groups using the Mann–Whitney U test. For comparing more than two groups, we used the Kruskal–Wallis test, followed by Bonferroni post hoc analysis. Correlations were evaluated using Spearman’s rank correlation test, with Spearman’s Rho and *p*-values reported in both graphs and the text.

Categorical variables were expressed as the number and percentage of patients, and the chi-squared test or Fisher’s exact test was applied when appropriate.

A univariate and multivariate logistic regression analysis was performed using the Facial Injury Severity Scale (FISS), dichotomized at its median value (FISS = 2), as the dependent variable (FISS ≤ 2 vs. FISS > 2). The following independent variables were included in the models: sex, age, presence of facial wounds, and number of fractures (single vs. multiple). Fracture pattern was categorized as a binary variable: single fracture, defined as involvement of a single anatomical site, and multiple fractures, defined as multiple associated fractures involving two or more distinct facial regions. Odds ratios (OR) with 95% confidence intervals (CIs) and *p*-values were calculated for each variable.

Statistical analyses were performed using IBM SPSS Statistics for Windows, version 29.0.1.0 (IBM, Armonk, NY, USA), and graphs were generated with GraphPad Prism 10.0 (GraphPad Software, Inc., La Jolla, CA, USA). A *p*-value < 0.05 was considered statistically significant. All statistical procedures followed best practices for epidemiological studies, ensuring robustness and reproducibility. Data analysis was conducted in compliance with guidelines for reporting observational studies in epidemiology.

## 3. Results

The study included 224 patients, who were victims of IPV between 2011 and 2022. The majority were male (94.2%), with a median age of 26 (22–34) years. Mandibular fractures were observed in 94 patients (42%), whereas 25% of cases involved orbital fractures. The remaining patients presented with zygomaticomaxillary complex injuries or multiple associated fractures (Table 1).

**Figure 1:**
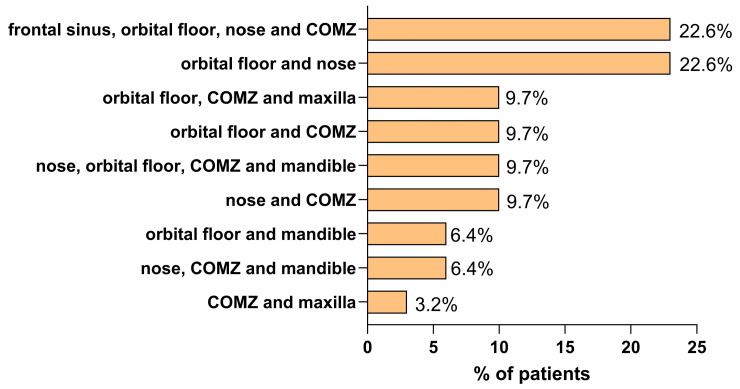
Distribution of fracture associations in patients with injuries from interpersonal violence. The bar chart shows the percentage of patients presenting with various combinations of fracture sites. The most frequent associations involved the frontal sinus, orbital floor, nose, and zygomaticomaxillary complex (COMZ), with 23%, and the orbital floor and nose, with 23%.

Among patients with multiple associated fractures, the most common combination involved fractures of the orbital floor and nasal bone alone (22.6% of patients) or in combination with fractures of the frontal sinus and zygomaticomaxillary complex (22.6%) (Figure 1).

Most patients with mandible injuries exhibited mandibular angle fractures (41.4%). Regarding condylar fractures, 58.3% were in the subcondylar region, whereas 41.7% involved other portions of the condyle.

Orbital fractures primarily affected the orbital floor (62.35%); 25.9% involved both the floor and the medial wall of the orbit. As shown in Table 2, among the seven patients with frontal sinus injuries, five had a fracture of the frontal wall. Moreover, 80% of the five patients with maxillary fractures presented a type I Le Fort fracture.

In 4 of the 224 patients, other associated injuries were recorded, such as skull or rib fractures. Among all the patients, 20 (8.9%) presented with facial soft-tissue wounds, which were further classified based on size. Specifically, 14 patients (70%) had wounds <10 cm, whereas 6 patients (30%) had wounds >10 cm. The mean FISS was 2.1 ± 1.6. Most patients underwent open reduction and internal fixation (ORIF) surgery after at least 24 h from hospital admission, with a mean hospitalization length of 4.1 ± 1.6 days.

**Figure 2:**
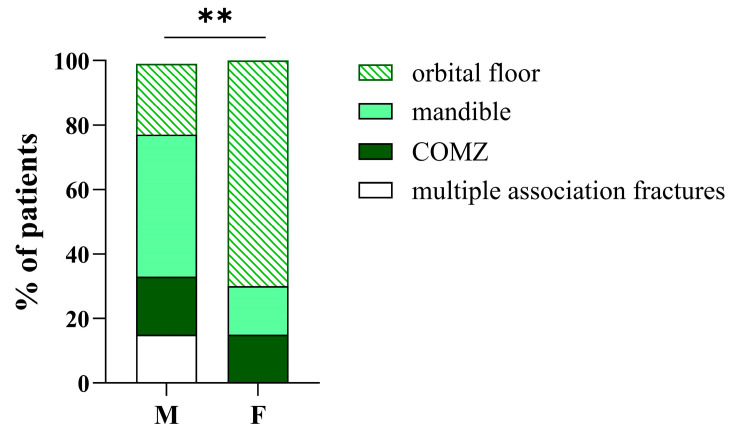
Sex-based distribution of fracture locations in patients with injuries from interpersonal violence. Stacked bar plots represent the percentage of male (M, *n* = 211) and female (F, *n* = 13) patients, according to fracture location. The bars are segmented into four categories: orbital floor, mandible, zygomaticomaxillary complex (COMZ), and multiple associated fractures. ** = *p* < 0.01.

When analyzing fracture sites by sex, we observed a difference between male and female patients. Specifically, the mandible was the most common fracture site among males (43.6%), whereas the orbital floor was most frequently affected in females (69.2%) (*p* < 0.01) (Figure 2). However, due to the small number of female patients in our cohort (*n* = 13), this finding should be interpreted with caution, and further studies are needed to confirm this observation.

**Figure 3:**
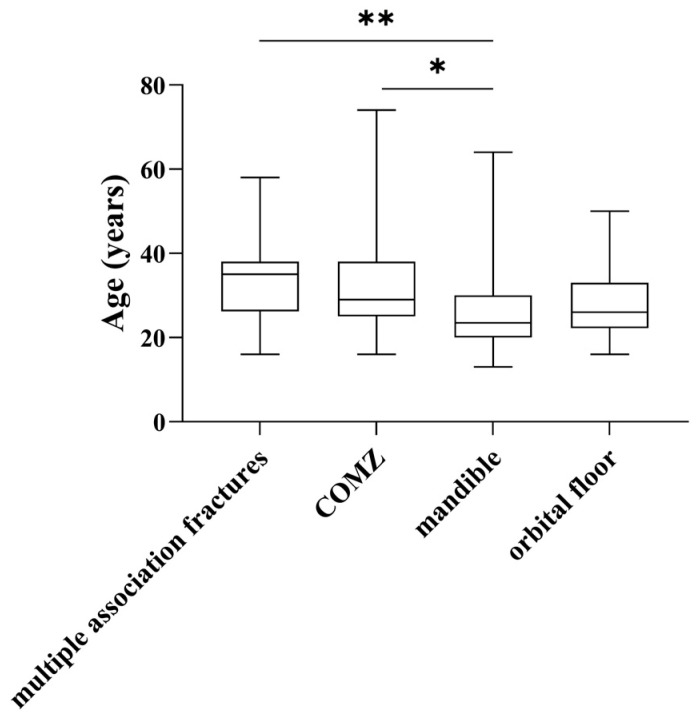
Age distribution of patients with fractures. The box plots represent the median and interquartile range of ages. * = *p* < 0.05, ** = *p* < 0.01.

In addition, we observed that patients with mandibular fractures were generally younger than those with zygomaticomaxillary complex fractures (23.5 (10) vs. 29.0 (13), respectively, *p* < 0.05) or multiple associated fractures (23.5 (10) vs. 35.0 (12), respectively, *p* < 0.01) (Figure 3).

We also observed an association between mandible fractures and face wounds. Although facial soft-tissue wounds were not frequently encountered, they were mostly found in patients with mandibular fractures, compared with those with other types of injuries, as shown in Table 3.

**Figure 4:**
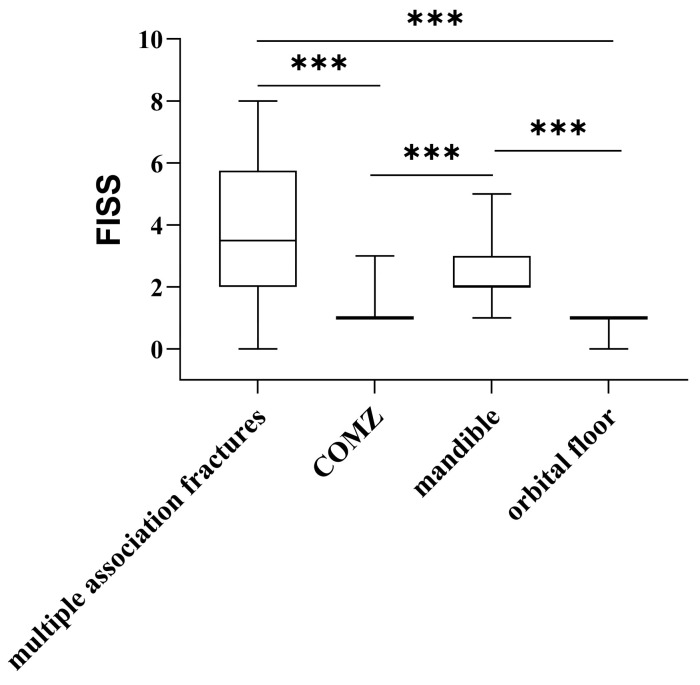
FISS scores according to anatomical site of fracture. Box plots show Facial Injury Severity Scale (FISS) scores across different fracture patterns: mandibular, zygomaticomaxillary complex (COMZ), orbital floor, and multiple fractures. The plot displays the median and interquartile range for each group. Patients with multiple fractures exhibited the highest FISS scores. A statistically significant difference was observed among the groups. *** = *p* < 0.001.

Notably, a lower FISS was detected in patients with orbital floor and zygomaticomaxillary complex fractures, compared with those with mandibular and multiple associated fractures (*p* < 0.001; Table 4 and Figure 4).

Evaluating FISS, we found that it was higher in male than in female patients (*p* < 0.01) and in older (>34 years) than in younger patients (*p* < 0.05). Moreover, patients with facial soft-tissue wounds had a higher FISS, compared to those without wounds (*p* < 0.001) (Table 4).

To further explore the factors associated with higher FISS scores, a univariate and multivariate logistic regression analysis was performed, using FISS > 2 as the outcome (Table 5). In the univariate analysis, the presence of facial wounds (*p* = 0.021) and having multiple fractures (*p* < 0.001) were significantly associated with FISS > 2. These associations remained significant in the multivariate model, where facial wounds (OR 3.1, 95% CI 1.2–8.1, *p* = 0.024) and multiple fractures (OR 0.3, 95% CI 0.1–0.6, *p* < 0.001) were identified as independent predictors. Age and sex were not significantly associated with higher FISS scores in either model.

**Figure 5:**
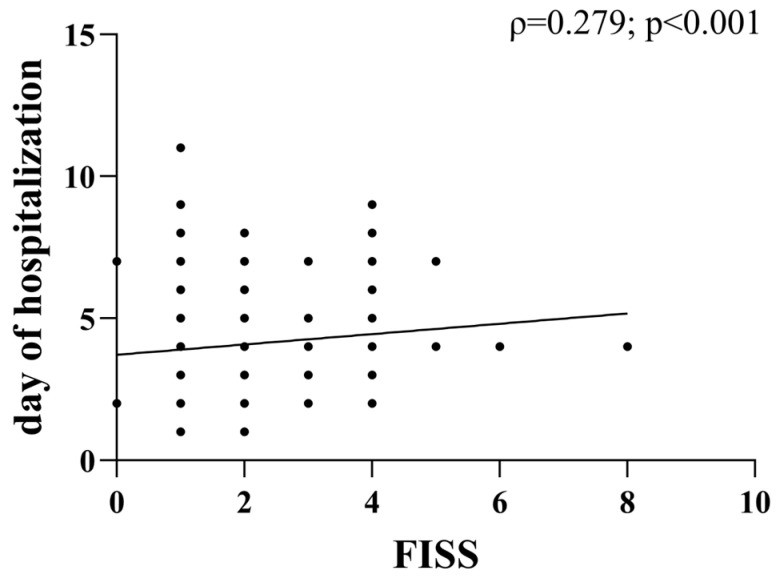
Severity of facial trauma is associated with longer hospitalization. The scatter plot illustrates the correlation between the number of hospitalization days and FISS.

We also noted a weak but significant positive correlation between FISS and the number of days of hospitalization (ρ = 0.23, *p* < 0.001) (Figure 5).

**Figure 6:**
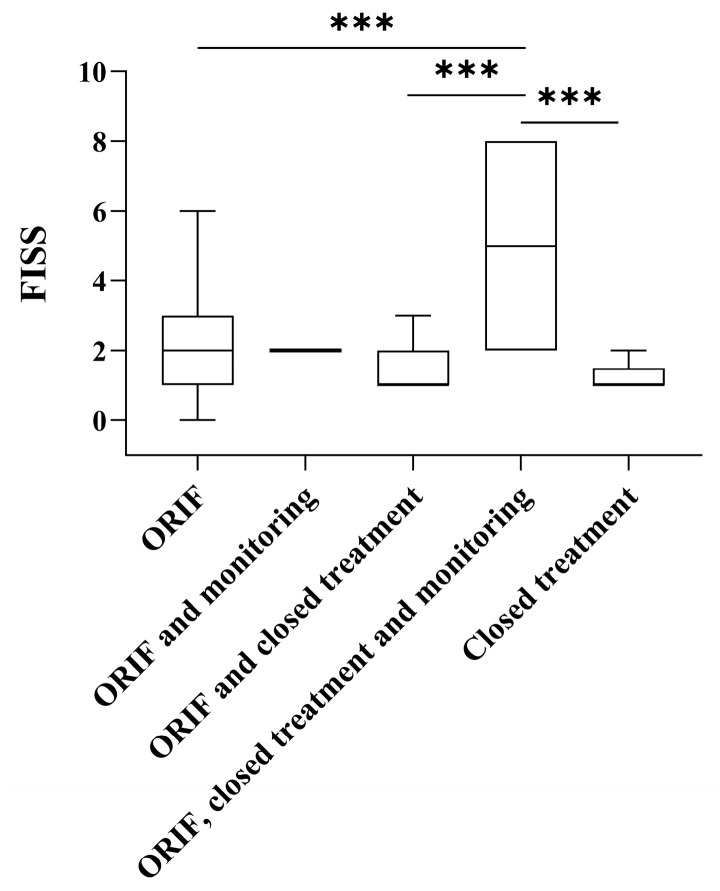
Facial Injury Severity Scale (FISS) scores in relation to treatment approach. Box plots display the distribution of FISS scores across different treatment groups. Each box represents the interquartile range (IQR), with the horizontal line indicating the median value. Statistically significant differences were observed between treatment groups. *** = *p* < 0.001.

This correlation highlights the importance of early and targeted interventions to optimize resource allocation in trauma care. Finally, we found FISS to be higher in patients who received combination treatment, including ORIF, closed treatment, and observation, compared with all other treatment groups (Figure 6).

### Trends in Facial Trauma

Over the 12-year period 2011–2022, a total of 224 admissions for interpersonal violence facial fractures were recorded, corresponding to a mean of 18.7 cases per year. When grouped into three consecutive four-year blocks, the caseload was 71 (2011–2014), 72 (2015–2018), and 81 (2019–2022), an absolute increase of 10 cases (+14.1%) between the first and last block. The corresponding mean annual numbers were 17.75, 18.00, and 20.25 cases. Using a constant catchment population of 1.48 million, the block-specific mean annual incidences were 1.20, 1.21, and 1.36 per 100,000 inhabitants, yielding an overall average annual incidence of 1.26 per 100,000. The block-to-block change equates to an estimated average annual growth of approximately 1.6%.

## 4. Discussion

Our study confirmed that IPV remains a primary cause of maxillofacial trauma, particularly among young males, with a male-to-female ratio of 16.23:1. This ratio aligns with the literature, which reports similar demographic trends globally [2,35,36]. The predominance of male victims is often attributed to their higher involvement in social activities that place them at increased risk of violent confrontations, particularly in urban settings, where alcohol consumption and nightlife activities contribute to heightened aggression [36].

The median age of 26 years is also consistent with findings from other studies, indicating that younger individuals, particularly men, are at higher risk of IPV-related injuries, owing to increased social exposure and engagement in high-risk behaviors [37,38,39].

The frequent involvement of the mandible may be attributed to its prominence and mechanical vulnerability during physical altercations. Studies have indicated that the angle and condyle of the mandible are particularly prone to fracture, owing to the force distribution during direct impact [40,41,42,43]. Meanwhile, we observed that orbital fractures were the second most common injury type, a finding that differs from other reports that ranked zygomatic fractures higher [44]. This discrepancy highlights the need for further regional analyses to elucidate injury patterns and their correlation with assault mechanisms.

A notable aspect of our findings is the weak but significant correlation between the Facial Injury Severity Score (FISS) and hospitalization length (ρ = 0.23, *p* < 0.001). Patients with higher FISS scores required longer hospital stays, reinforcing the score’s relevance as a prognostic indicator for trauma severity and resource allocation in healthcare settings. Additionally, we found that males had significantly higher FISS scores compared to females (*p* < 0.01), and older patients (>34 years) had more severe injuries than younger ones (*p* < 0.05). However, due to the small number of female patients, this sex-related difference should be interpreted with caution and will require further confirmation. These findings suggest that IPV-related injuries in older individuals may be more severe due to greater force involved in the trauma or delayed healthcare access.

Our results also indicate that patients with mandibular fractures were more likely to have facial soft-tissue wounds, compared to those with other fracture types (*p* < 0.001). This observation underscores the need for early wound management to prevent complications such as infection and soft-tissue necrosis [45].

To better understand which factors were independently associated with higher trauma severity, we performed a multivariate logistic regression, using FISS > 2 as the outcome. The analysis confirmed that the presence of facial soft-tissue wounds and multiple fractures were significant predictors of increased injury severity, even after adjusting for age and sex. These findings highlight the prognostic value of both external injuries and fracture complexity in the acute management of IPV-related maxillofacial trauma.

A significant proportion of patients underwent ORIF within 24 h, primarily among those with mandibular fractures and visible facial soft-tissue wounds (*p* < 0.05). This suggests that visible external injuries may lead to expedited surgical decision-making, highlighting the potential influence of clinical presentation on treatment prioritization. Moreover, external injuries may influence early surgical intervention decisions, emphasizing the importance of a standardized triage protocol for IPV-related maxillofacial trauma [46].

Our 12-year single-center series is consistent with the demographic core reported across maxillofacial trauma studies, in which young males are systematically over-represented among assault victims [15]. The proportion of facial fractures attributed to interpersonal violence in Italy varies by geography and study design, spanning roughly 13–30% of all maxillofacial fractures: lower shares in some central provinces, with a higher burden of road traffic accidents [47] and higher shares in large southern urban series [48].

The anatomical distribution in our cohort (mandible first, orbit second) partly mirrors national patterns but also shows comparatively higher orbital involvement. In the EURMAT prospective collaboration, mandibular fractures comprised about 38% of assault-related facial fractures, and orbital fractures ≈14% [15]; the Turin assault series similarly reported mandible 34% and orbit 9% [16], whereas the large Naples cohort (mixed etiologies, 2001–2015) recorded mandible 35.4% and zygomaticomaxillary complex ≈29.9% [48]. By contrast, the Terni (Umbria) series found a predominance of orbital floor involvement (≈39%) over zygoma (≈26%) and mandible (≈20%) [47], highlighting regional variability that likely reflects differences in prevailing mechanisms (closed-fist nightlife altercations vs. mixed lower-energy impacts) and imaging thresholds. Our relatively elevated orbital percentage aligns more closely with central Italian patterns, where low- to moderate-energy blows and mixed assaults produce frequent orbital floor or medial wall fractures while still retaining the mandibular predominance typical of punch injuries.

Temporal dynamics reported nationally emphasize a progressive reduction in the proportion of road traffic accident (RTA)-related facial fractures and a stable or modestly rising share of assaults over the last decade [14,49]. Pandemic restrictions acted as a natural “interruption” in these trajectories: multiple Italian datasets document a sharp 2020 contraction in total facial fracture admissions, with a relative increase in domestic or interpersonal episodes and a fall in RTA incidence, followed by a rebound toward pre-pandemic volumes in 2021–2022 [50,51,52]. Against this national backdrop, our block-wise caseload rise (+14.1% from 2011–2014 to 2019–2022) represents a modest incremental increase rather than a structural surge, consistent with a plateauing but persistent endemic burden of interpersonal violence that existing road safety policies do not directly influence.

Socioeconomic and demographic factors further modulate the Italian pattern: southern multicenter experiences and socioeconomic analyses report higher assault proportions among foreign-born or socially disadvantaged subgroups and link uneven regional development to variability in interpersonal violence exposure (e.g., influence of socioeconomic factors study). Such disparities help explain the geographically stratified assault shares and reinforce the need for context-sensitive preventive strategies.

In synthesis, three comparative points emerge: (i) our local assault burden sits within the Italian mid-to-upper range of published proportions, confirming the sustained clinical workload; (ii) the mandibular predominance with relatively elevated orbital involvement places our anatomical pattern between northern (lower orbital share) and central cohorts with higher orbital floor representation; and (iii) the modest upward block-wise caseload, together with pandemic perturbations seen nationally, underscores the resilience of interpersonal violence as a driver of facial fractures and the opportunity for targeted primary prevention (alcohol/nightlife harm reduction, early assault risk screening, socioeconomic vulnerability interventions) complementary to already effective RTA mitigation policies.

In Southern Europe, a progressive reduction in road traffic accident (RTA)-related facial injuries has coincided with the enforcement of stricter seat belt and helmet laws, while interpersonal violence (IPV) has emerged as the leading cause among young adults [1,41,53]. This finding underscores the persistent and systemic nature of IPV, which continues to generate a non-negligible clinical and economic workload for specialized trauma services. A similar trend has been reported in other European studies, suggesting that existing preventive strategies have been largely ineffective in reducing IPV-related trauma [21]. Alcohol and drug misuse amplify this trend: hospital series show that assault-related facial fractures peak at weekends and are strongly linked to intoxication. Age and sex further modulate fracture patterns—men in their twenties sustain high-energy mandibular or zygomatic fractures, whereas women, often assaulted by partners, present more frequently with orbital injuries [16]. The lack of a downward trend highlights the need for more effective preventive policies, including public health campaigns, educational programs, and community interventions aimed at reducing violent behavior and promoting conflict resolution strategies [54].

Nearly one-half of patients with maxillofacial fractures carry at least one serious associated injury, and one-third develop neurological deficits, most commonly facial nerve or infra-orbital paresthesia [55,56]. Infection and malocclusion remain the main postoperative complications, with demographic and modifiable factors (smoking, delayed fixation) significantly affecting outcome metrics. Beyond physical morbidity, complex facial trauma imposes substantial quality-of-life deterioration and economic loss through prolonged sick leave and repeated surgical care [57,58].

Improving triage and subsequent management in IPV-related head and neck injuries is paramount to reducing morbidity and enhancing both functional and aesthetic outcomes. First, a standardized screening protocol—involving maxillofacial surgeons, emergency physicians, radiologists, and psychologists—could facilitate prompt detection of severe fractures and associated mental health issues. Second, validated tools like FISS may guide early surgical decisions, prioritize operative intervention, and predict resource utilization. Third, immediate and meticulous repair of extensive facial soft-tissue wounds is essential not only to prevent infections but also to preserve aesthetic contours and patient self-esteem. Finally, a structured postoperative follow-up, encompassing functional and psychological assessments, is critical to ensure comprehensive recovery and detect late complications that may compromise aesthetic and functional outcomes. Although facial soft-tissue wounds were infrequent (8.9%), their presence almost doubled the probability of an underlying mandibular fracture (OR = 2.6, 95% CI 1.3–5.1; *p* = 0.005). Similar figures have been reported in larger multicentric series, where one-third to two-thirds of patients with facial fractures also show soft-tissue injuries, especially after high-energy trauma [59]. Recognizing this association in the acute setting is crucial: even a minor laceration in the lower third of the face should lead to mandibular CT execution to avoid missed fractures and to guide debridement and fixation strategies.

Despite its retrospective design, this study offers several strengths. First, our 12-year observation period provides a broad temporal landscape of IPV-related facial trauma within a single regional center. Second, the use of standardized classification criteria (AO-CMF) and severity scoring (FISS) enhances data reliability and comparability. Lastly, by focusing on IPV as an etiology, our findings contribute crucial insights that may support targeted prevention and resource allocation strategies in central Italy and similar contexts.

### Study Limitations

As with all retrospective studies, this research is subject to information and selection biases linked to the quality of medical records. First, our database did not systematically capture contextual risk factors that are well recognized in IPV-related trauma—namely, alcohol or illicit drug intoxication, the precise mechanism of assault (blunt versus sharp/penetrating force), and the exact timing of injury (hour of day, weekday vs. weekend). Their absence precluded any sub-analysis of these variables and may conceal important epidemiological signals or introduce residual confounding. Second, some patients might have concealed the true circumstances of their injuries, leading to under-ascertainment of IPV cases. Third, certain fracture sites (e.g., nasal fractures managed in otorhinolaryngology wards) were excluded, potentially underestimating the full burden of IPV-related facial trauma. Furthermore, data on the day of the week or time of injury were rarely documented, preventing an analysis of potential temporal patterns. Fourth, we observed no significant decrease in IPV-related admissions over the 12-year period, suggesting that existing preventive measures may be insufficient. Future studies should evaluate the effectiveness of public health initiatives and law enforcement strategies to reduce IPV-related maxillofacial trauma. Fifth, although our hospital covers a catchment population of approximately 1.4 million inhabitants, only 224 admissions for IPV-related facial fractures were captured between 2011 and 2022 (mean 18.7 cases yr^−1^). Several mechanisms inevitably drive case under-ascertainment: (i) minor facial injuries or isolated nasal/dento-alveolar fractures managed in outpatient settings were excluded by design; (ii) victims frequently withhold information on assault, resulting in miscoding at first presentation; (iii) incomplete screening by emergency staff, who documented that most OMFS clinicians felt only ‘somewhat confident’ in recognizing IPV; and (iv) the absence of a regional trauma registry specifically for interpersonal violence. Collectively, these aspects suggest that the true burden of IPV-related facial trauma is underestimated. These small numbers reflect both the rarity of hospital-admitted fractures, relative to all IPV events, and the well-recognized phenomenon of under-reporting. Finally, the strong imbalance in sex distribution within our sample (211 males vs. 13 females) introduced a condition of complete separation in the logistic regression model, whereby none of the female patients had a FISS score >2. As a result, the odds ratio for female sex could not be reliably estimated and should not be interpreted as evidence of a true protective effect. This statistical artifact reflects the low representation of women in our cohort and further highlights the need for more inclusive sampling in future research to allow for robust subgroup analyses.

## 5. Conclusions

Our study provides valuable epidemiological insights into IPV-related maxillofacial fractures in central Italy, emphasizing the need for targeted prevention strategies and efficient healthcare resource allocation. By demonstrating the persistent nature of IPV-related injuries, our findings highlight the necessity of multidisciplinary intervention approaches, including law enforcement collaboration, public awareness campaigns, and expanded mental health support for victims. The findings reinforce the importance of early intervention, standardized treatment protocols, and multidisciplinary approaches to addressing IPV-related trauma. Future research should focus on identifying risk factors for IPV-related injuries, evaluating the effectiveness of preventive programs, and implementing standardized data collection methods to improve trauma reporting and patient outcomes.

## Figures and Tables

**Table 1:** Distribution of epidemiological, clinical, and surgical data in the study cohort.

	N = 224
Gender—*n* (%)	
Female/Male	13/211 (5.8/94.2)
Age	
Mean ± SD	29.1 ± 10.5
Median (Q1–Q3)	26.0 (22.0–34.0)
Fracture Site—*n* (%)	
Association of multiple fractures	32 (14.3)
Zygomaticomaxillary complex	41 (18.3)
Mandible	94 (42.0)
Maxilla	1 (0.4)
Nose	31 (13.8)
NOE	6 (2.7)
Orbital Region	56 (25.0)
Frontal sinus	7 (3.1)
Face wounds—*n* (%)	
Yes	20 (8.9)
No	204 (91.1)
FISS	
Mean ± SD	2.1 ± 1.6
Median (Q1–Q3)	2 (1–3)
Timing of surgery—*n* (%)	
<24 h	13 (5.8)
24–72 h	100 (44.6)
>72 h	111 (49.6)
Day of hospitalization	
Mean ± SD	4.1 ± 1.6
Median (Q1–Q3)	4.0 (3.0–5.0)
Type of treatment—*n* (%)	
ORIF	174 (77.7)
ORIF and monitoring	5 (2.2)
ORIF and closed treatment	17 (7.6)
ORIF, closed treatment, and monitoring	15 (6.7)
Monitoring	0 (0.0)
Closed treatment	13 (5.8)

Abbreviations: FISS = Facial Injury Severity Score, N = Number of patients, NOE = Naso–orbito–ethmoid, ORIF = Open reduction and internal fixation, Q1 = First quartile, Q3 = Third quartile, SD = Standard deviation. Fracture numbers include fractures occurring either in isolation or in combination with other sites.

**Table 2:** Fracture site classification according to Arbeitsgemeinschaft für Osteosynthesefragen surgery reference.

	N (%)
Fracture of Mandible	
N	101
Angle of the mandible	41 (40.6)
Condyle	3 (3.0)
Body of the mandible	2 (2.0)
Dentoalveolar	8 (8.0)
Symphysis	1 (1.0)
Angle and condyle	1 (1.0)
Angle and body	6 (6.0)
Angle and symphysis	9 (9.0)
Angle and parasymphysis	9 (7.0)
Condyle and body	12 (11.9)
Condyle and symphysis	1 (1.0)
Condyle and parasymphysis	7 (7.0)
Body and parasymphysis	1 (1.0)
Fracture of orbital region	
N	85
Orbital floor	53 (62.4)
Medial wall of the orbit	2 (2.4)
Roof of the orbit	1 (1.2)
Orbital floor and lateral wall	7 (8.2)
Orbital floor and medial wall of the orbit	22 (25.9)
Fracture of frontal sinus	
N	7
Anterior and back part	2 (28.6)
Frontal wall	5 (71.4)
Fracture of maxilla	
N	5
LE FORT I	4 (80.0)
LE FORT II	1 (20.0)

Abbreviation: N = Number of patients. Data refer to 198 out of 224 total patients.

**Table 3:** Association of fracture and face wound in the study cohort.

Fracture Site	Face Wounds, *n* (%)		
	Yes	No	*p*-Value
Multiple associated fractures	3 (15.0)	29 (14.2)	0.005
Zygomaticomaxillary complex	2 (10.0)	39 (19.1)	
Mandible	15 (75.0)	79 (38.7)	
Maxilla	0 (0.0)	1 (0.5)	
Orbital region	0 (0.0)	56 (27.5)	

Abbreviation: N = Number of patients. *p*-value obtained with chi-squared test.

**Table 4:** FISS score.

	FISS Score	*p*-Value
Gender		
Female	1.0 (1.0–1.0)	0.005 *
Male	2.0 (1.0–3.0)	
Age		
≤34 years	2.0 (1.0–2.5)	0.035 *
>34 years	2.0 (1.0–4.0)	
Fracture Site		
Association	3.5 (2.0–5.8)	<0.001 ^+^
Zygomaticomaxillary complex	1.0 (1.0–1.0)	
Mandible	2.0 (2.0–3.0)	
Maxilla	2.0 (2.0–2.0)	
Orbital region	1.0 (1.0–1.0)	
Face wounds		
Yes	2.5 (2.0–4.0)	<0.001 *
No	1.5 (1.0–2.75)	
Timing of surgery		
<24 h	2.0 (1.0–2.5)	0.675
24–72 h	1.0 (1.0–3.0)	
>72 h	2.0 (1.0–3.0)	
Type of treatment		
ORIF	2.0 (1.0–3.0)	<0.001 ^+^
ORIF and monitoring	2.0 (2.0–2.0)	
ORIF and closed treatment	1.0 (1.0–2.0)	
ORIF, closed treatment and monitoring	5.0 (2.0–8.0)	
Closed treatment	1.0 (1.0–1.5)	

Abbreviations: FISS = Facial Injury Severity Score, ORIF = Open reduction and internal fixation. FISS was represented as median (first quartile–third quartile). * *p*-value obtained by Mann–Whitney test; ^+^ *p*-value obtained by Kruskal–Wallis test, followed by Bonferroni post hoc analysis.

**Table 5:** Univariate and multivariate logistic regression analysis for risk of having a Facial Injury Severity Scale (FISS) > 2.

	Univariate				Multivariate			
Variables	*n*	OR	95% CI	*p*-Value	*n*	OR	95% CI	*p*-Value
Sex								
F vs. M	224	0.00	0.00	0.999	224	0.00	0.00	0.999
Age								
>34 vs. ≤34 years	224	1.7	0.8–3.2	0.143	224	1.3	0.6–2.6	0.568
Type of fracture								
Single vs. Multiple	224	0.2	0.1–0.5	<0.001	224	0.3	0.1–0.6	<0.001
Face wounds								
Yes vs. No	224	3.0	1.2–7.6	0.021	224	3.1	1.2–8.1	0.024

Abbreviations: OR = Odds ratio, IC = Interval of confidence.

## Data Availability

The data generated and analyzed during this study are not publicly available due to institutional and privacy policies but are available from the corresponding author upon reasonable request.
